# A Rare Case of Trigger Finger Caused by Tumoral Calcinosis in Zone III of the Palm

**DOI:** 10.7759/cureus.100721

**Published:** 2026-01-04

**Authors:** Masashi Sato, Tetsuya Nakatani, Tetsuro Kurashina, Takafumi Onga

**Affiliations:** 1 Orthopaedic Surgery, Nakatani Orthopaedic Surgery Hospital, Kakogawa, JPN

**Keywords:** calcium deposition, case report, hand, trigger finger, tumoral calcinosis, zone iii

## Abstract

Tumoral calcinosis (TC) is a pathological condition characterized by tumor-like calcific deposits in the soft tissues surrounding the joints. This condition typically affects large joints; however, TC involving hand tendons is extremely rare. There are no previously reported cases of TC occurring in Zone III of the palm, and no prior reports of trigger finger secondary to TC. We present a case of TC arising in Zone III of the palmar aspect of the right ring finger in a 65-year-old man who was referred for treatment of trigger finger. Surgical excision of the lesion resulted in symptomatic improvement, and no recurrence was observed seven months postoperatively. This case highlights the potential for atypical Zone III lesions to present as trigger finger and underscores the importance of considering space-occupying lesions in the differential diagnosis. It further emphasizes the need for careful palpation, ultrasonographic evaluation, and radiographic assessment in patients presenting with trigger finger.

## Introduction

Tumoral calcinosis (TC) is a pathological condition characterized by the deposition of calcium salts in the soft tissues surrounding joints. Clinically, patients with TC present with periarticular swelling, discomfort, pain, and restricted joint motion, most commonly affecting the hips, elbows, knees, and shoulders. Since its initial description by Duret in 1899, over 250 cases have been documented. However, TC involving the hand is extremely rare, and to our knowledge, no cases involving the palmar region (Zone III) have been reported [1-3].

Trigger finger is a multifactorial disorder characterized by painful popping or clicking during flexion and extension. While the condition is typically idiopathic, secondary trigger finger due to space-occupying lesions has also been documented [4,5]. Although TC can occur in distal extremities, it has not previously been reported as a cause of trigger finger, particularly not in Zone III of the palm. Furthermore, no prior reports have described the biomechanics by which a space-occupying lesion in Zone III, such as TC, can interfere with flexor tendon excursion or pulley function. Thus, we aimed to present a rare case of trigger finger caused by TC arising in the palmar region (Zone III).

## Case presentation

**Figure 1:**
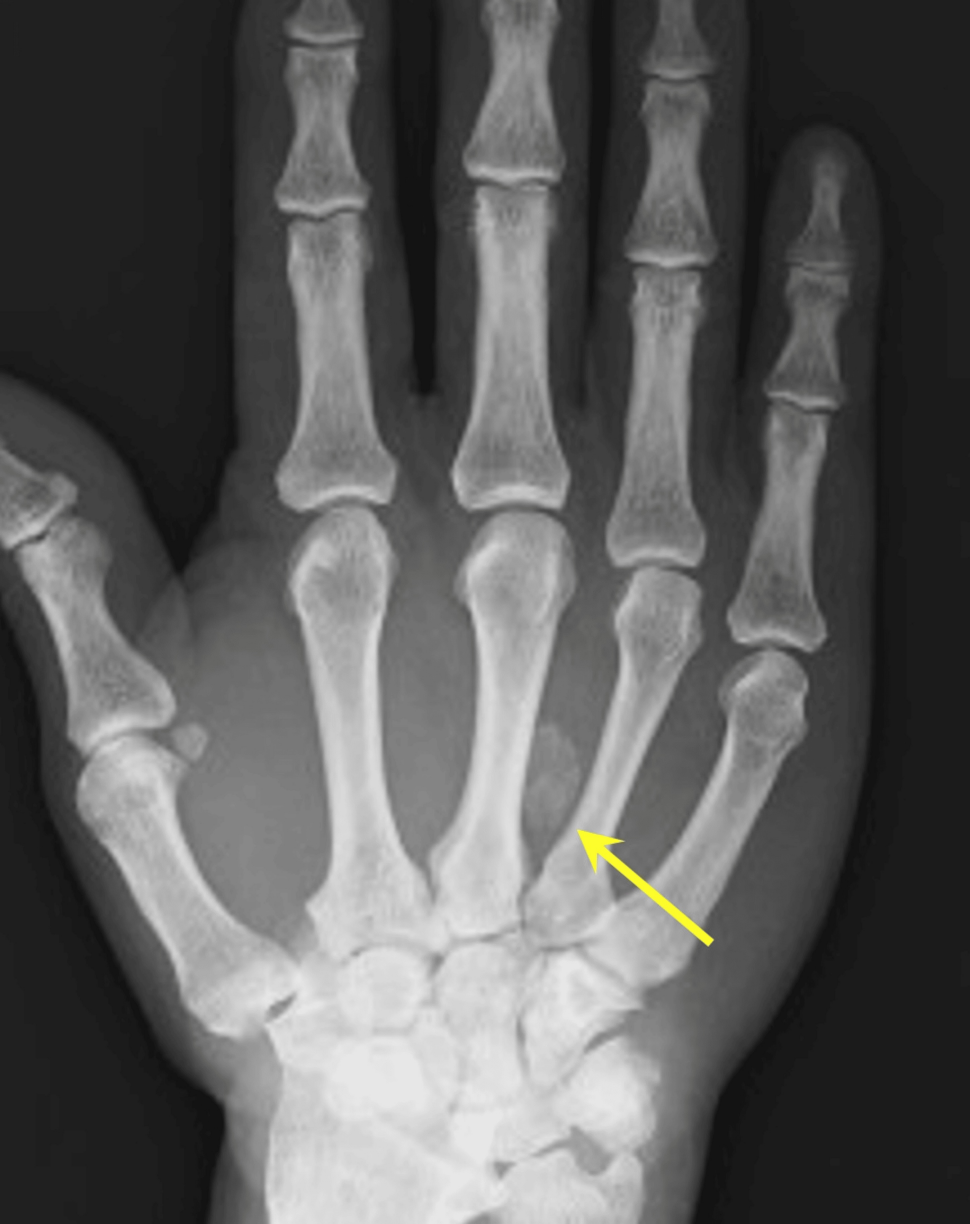
Preoperative radiograph A radiograph of the right-hand PA view shows a calcified mass between the third and fourth metacarpal bones (yellow arrow).

A 65-year-old right-handed man presented with a four-year history of pain in his right ring finger. The pain had no apparent precipitating cause and gradually worsened, leading to an increasing frequency of pain episodes and triggering. He reported no significant history of trauma, gout, diabetes, or family history of metabolic or calcific disorders. On physical examination, no tenderness over the volar aspect of the metacarpophalangeal joint was detected, and no visible swelling was observed in the palm. However, mild tenderness and a palpable mass were noted in the palmar aspect of Zone III, with reproducible triggering of the right ring finger present. No sensory deficits were observed. Plain radiography revealed a calcified lesion located between the metacarpal bones of the middle and ring fingers in the right palm (Figure 1).

**Figure 2:**
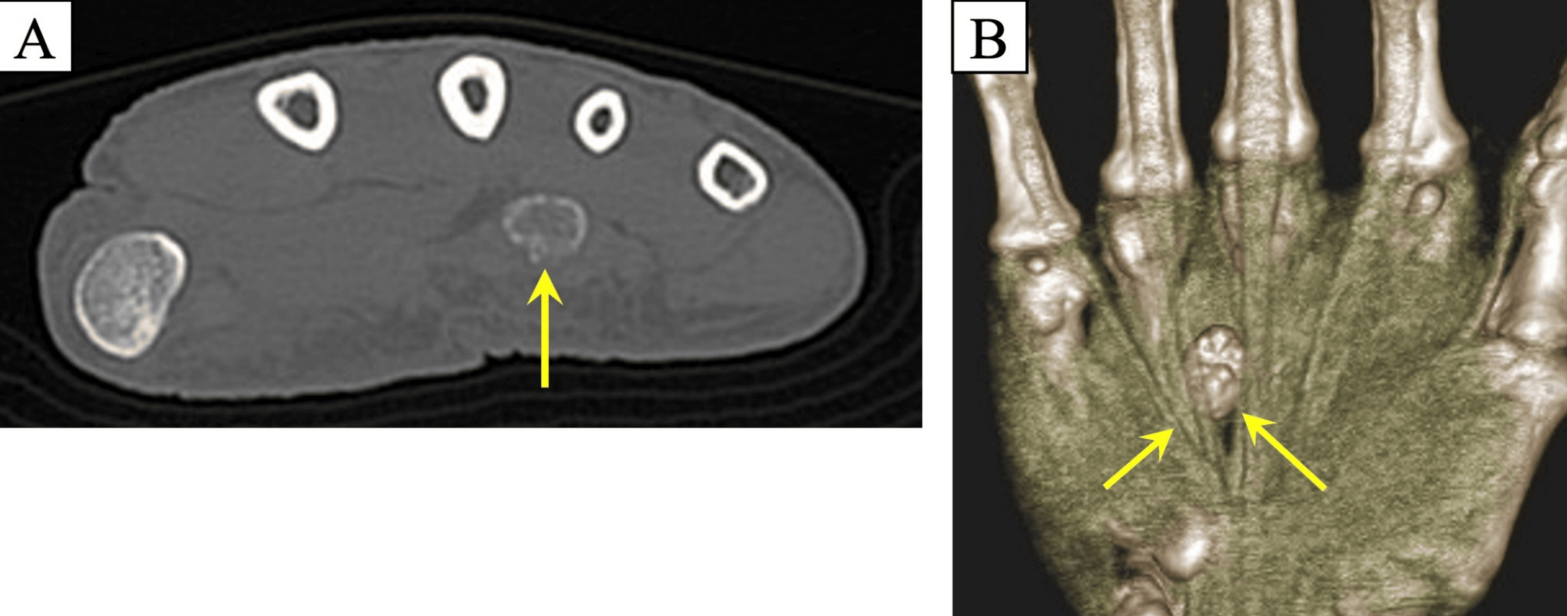
Preoperative computed tomography (A) Computed tomography of the right-hand axial view shows a well-defined calcified lesion; (B) The flexor digitorum profundus (FDP) tendon shows ulnar deviation due to displacement by the calcified mass (yellow arrows).

Computed tomography (CT) demonstrated a well-defined mass measuring 10 × 14 × 8 mm, associated with the ulnar deviation of the flexor digitorum profundus (FDP) tendon (Figure 2).

**Figure 3:**
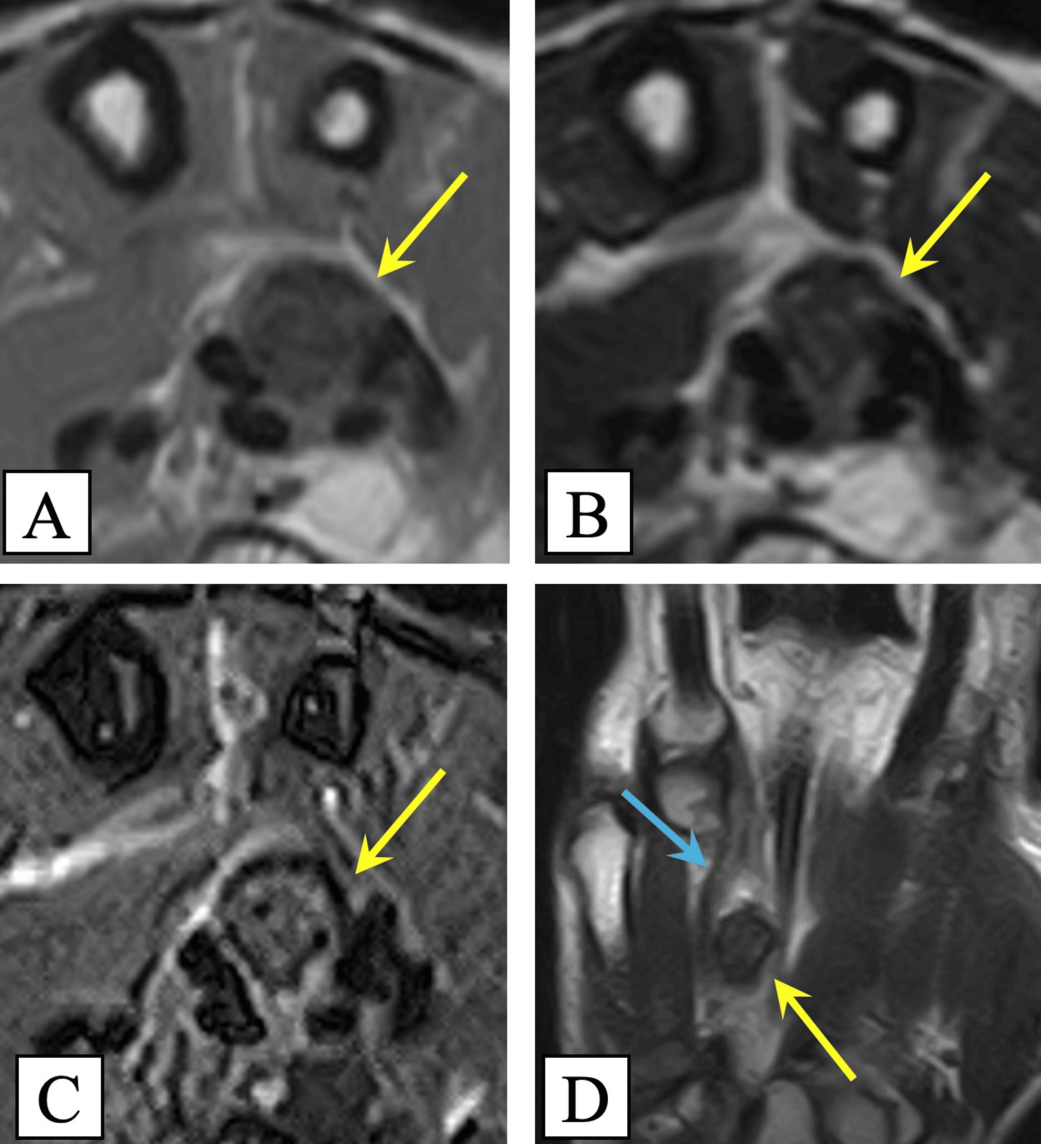
Preoperative magnetic resonance imaging (A) Axial T1-weighted image; (B) Axial T2-weighted image; (C) Axial short tau inversion recovery (STIR) image; (D) Coronal T2-weighted image. Magnetic resonance imaging reveals a well-defined lesion adjacent to the flexor digitorum profundus (FDP) tendon of the ring finger (blue arrow), showing low signal intensity on both T1- and T2-weighted images and heterogeneous high signal intensity with a mosaic-like appearance on the STIR image (yellow arrows)

Magnetic resonance imaging (MRI) revealed a lesion adjacent to the FDP tendon of the ring finger, characterized by smooth margins and displaying low signal intensity on both T1- and T2-weighted images. Additionally, heterogeneous high signal intensity with a mosaic-like pattern was observed on short tau inversion recovery image. The tortuosity of the FDP tendon was also evident (Figure 3).

**Figure 4:**
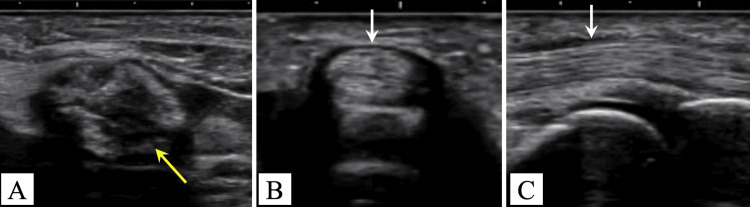
Preoperative ultrasonography (A) Ultrasonography shows a heterogeneous hyperechoic lesion between the third and fourth metacarpals, moving with finger motion (yellow arrow); (B, C) no thickening of the A1 pulley (white arrows).

Ultrasonography indicated no thickening of the A1 pulley of the ring finger. A heterogeneous hyperechoic lesion with distinct margins was observed between the middle and ring finger metacarpals, displaying movement in conjunction with active finger motion (Figure 4).

Laboratory investigations showed a mild decline in renal function, with an estimated glomerular filtration rate of 49.8 mL/min and a creatinine level of 1.16 mg/dL. Corrected calcium levels measured 9.0 mg/dL (reference range: 8.5-10.2), phosphorus levels were 2.7 mg/dL (reference range: 2.4-4.3), and intact parathyroid hormone levels were 65 pg/mL (reference range: 10-65), all of which were within normal limits (Table 1).

**Table 1:** Relevant blood study results undertaken on this patient All laboratory values were within normal ranges, except for the eGFR, which demonstrated a mild decline. PTH: parathyroid hormone

Test Study	Result	Reference Range	Comment
Creatinine (Cr)	1.16 mg/dL	0.7–1.3 mg/dL (adult male)	Within normal range
eGFR	49.8 mL/min/1.73 m²	≥ 90 normal; 60–89 mildly ↓; 45–59 mild–mod ↓	Mild decline
Corrected calcium	9.0 mg/dL	8.5–10.2 mg/dL	Within normal range
Phosphorus (P)	2.7 mg/dL	2.4–4.3 mg/dL	Within normal range
Intact PTH	65 pg/mL	10–65 pg/mL	Within normal range

**Figure 5:**
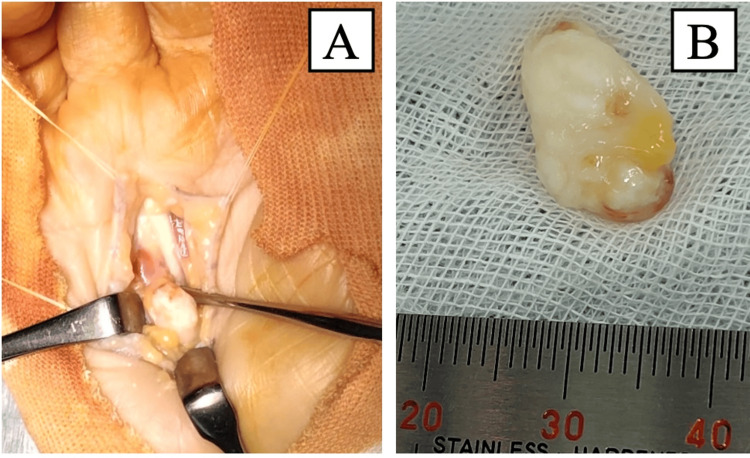
Intraoperative photographs (A) A smooth, firm, mobile, milky-white mass located deep to the flexor digitorum profundus (FDP) tendon, with partial adhesion to its radial aspect; (B) resected tumor

Given the presence of a distinct space-occupying lesion, conservative treatment was not considered prior to surgery; thus, surgical excision was performed. The lesion was excised under direct visualization while the patient was under general anesthesia. A zigzag incision approximately 6 cm in length was made on the right palm, and subcutaneous dissection revealed a firm, smooth, milky-white, and mobile mass adherent to the radial superficial aspect of the FDP tendon and partially attached to its tendon sheath (Figure 5).

**Figure 6:**
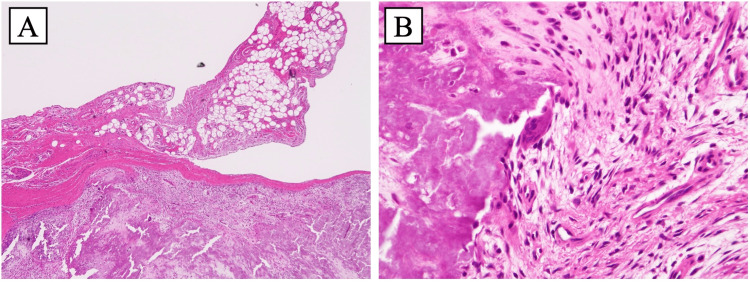
Histological sections of the surgical specimen. (A) 4× and (B) 40× magnification (hematoxylin and eosin staining) (A) Histological section shows a fibrous capsule surrounding diffuse basophilic calcium deposits and adjacent synovial-like structures. (B) Section shows multinucleated giant cells and inflammatory cells.

The lesion caused displacement and tortuosity of the FDP tendon, resulting in impaired gliding. Snapping persisted even after the incision of the palmar aponeurosis and was completely resolved only after the excision of the lesion. The tumor was excised en bloc, including the surrounding capsule and contents, which immediately eliminated passive triggering. Histopathological examination revealed a fibrous capsule surrounding diffuse basophilic calcium deposits, accompanied by multinucleated giant cells and focal synovial-like structures (Figure 6).

**Figure 7:**
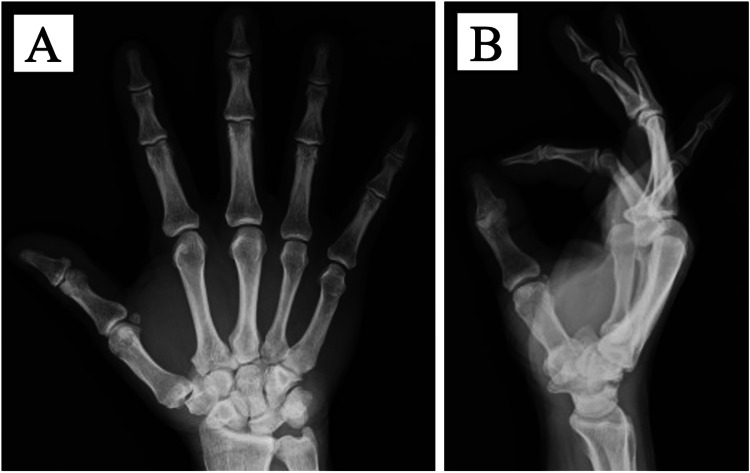
Postoperative radiographs acquired at seven months (A) anteroposterior view; (B) lateral view No recurrence of palmar calcification was noted on the radiographs

No external immobilization was implemented postoperatively. The triggering resolved immediately after surgery, and no limitation in the range of motion was observed. The patient underwent regular follow-up examinations, and at the final evaluation conducted 7 months postoperatively, no recurrence of symptoms or palmar calcification was noted on the radiographs (Figure 7).

## Discussion

TC is classified into three subtypes: primary normophosphatemic, primary hyperphosphatemic, and secondary [6]. Primary normophosphatemic TC is characterized by classic calcific lesions on imaging, a lack of history of disorders that cause soft tissue calcification, and normal serum phosphate and calcium levels [2]. In the present case, the absence of an underlying metabolic or systemic condition associated with calcification, along with normal phosphate and calcium levels, supports the diagnosis of primary normophosphatemic TC.

Radiographically, TCs typically present as amorphous, multilobulated, and calcified cystic masses surrounding the joints. CT provides clearer delineation of these lesions and often reveals well-defined calcified deposits without adjacent bone erosion or destruction. MRI generally demonstrates nodular lesions exhibiting heterogeneous low signal intensity on T1-weighted images and variable low-to-high signal intensity on T2-weighted images. Due to their diverse imaging features, TCs may mimic other causes of soft tissue calcification, such as heterotopic ossification, exostosis, or gouty tophi [7,8]. The MRI findings in this case were consistent with typical imaging features.

Triggering caused by TC affecting the fingers is extremely rare, with only a few cases reported in the literature. To our knowledge, this is the first reported case of TC in the palmar region (Zone III) of the hand [2]. Despite the lack of previous reports describing trigger finger caused by TC, this was classified as a secondary case of trigger finger resulting from a space-occupying lesion. Most previously reported secondary cases have involved lesions around the A1 or A2 pulleys [4]; however, in this case, imaging revealed that the lesion was located in Zone III, proximal to the A1 pulley.

Two hypotheses have been proposed to explain the mechanism underlying the triggering phenomenon associated with lesions in Zone III.

The first hypothesis suggests that a mechanical triggering effect arises from increased gliding resistance between the palmar aponeurosis and the flexor tendon. The palmar aponeurosis, situated proximal to the A1 pulley, may serve as a pulley. Wu et al. reported that the A0 pulley, which consists of the transverse aponeurotic fibers of the palmar aponeurosis, accounts for 31-47% of trigger finger cases [9,10], thereby supporting this concept. Consequently, mechanical interference between the palmar aponeurosis and the flexor tendon may have contributed to the triggering observed in this case.

The second hypothesis proposes that abnormal tendon gliding occurs due to the formation of a lesion between the flexor digitorum superficialis (FDS) and FDP tendons. Kazuki et al. described trigger finger as a condition resulting from an intertendinous connection between the FDS and FDP tendons, suggesting that impairment of normal tendon gliding can lead to the snapping phenomenon [11].

In the present case, however, the intraoperative findings contradicted the initial hypothesis: snapping persisted even after the incision of the palmar aponeurosis. Notably, triggering was completely resolved following the excision of the lesion. Although voluntary motion could not be assessed due to the absence of wide-awake surgery, which was not performed, these findings suggest that the lesion caused tortuosity of the FDP tendon, resulting in abnormal gliding between the FDS and FDP tendons and subsequent triggering. This observation supports the secondary hypothesis. Treatment should be guided by the classification, stage, location, size, and associated symptoms of the lesion, with early surgical excision being the preferred treatment option.

Conversely, in cases of hyperparathyroidism, effective medical management is essential, as an uncontrolled metabolic imbalance significantly increases the risk of recurrence. Secondary TC necessitates addressing the underlying metabolic disorders to achieve definitive control. In instances associated with hyperparathyroidism, parathyroidectomy is the preferred approach; the simple excision of a calcified mass alone is not recommended [3,7].

In the present case, a solitary normocalcemic TC developed in the finger, originating from the FDP tendon sheath. This lesion likely caused chronic mechanical friction on the flexor tendon, resulting in trigger finger. Considering both the local mechanical effects and the underlying pathology, surgical excision was performed and led to a favorable postoperative outcome without recurrence. This case report has certain limitations, which include the absence of wide-awake intraoperative dynamic assessment, a relatively short follow-up period (seven months), and the inability to directly assess preoperative tendon gliding beyond imaging.

## Conclusions

The present case report describes a rare instance of trigger finger caused by TC within Zone III of the right ring finger. When evaluating patients with trigger finger, clinicians should consider the possibility of space-occupying lesions, including atypical tendon calcifications, such as TC. A comprehensive diagnostic assessment utilizing plain radiography and ultrasonography is essential for identifying secondary causes. Accurate identification of the underlying pathology is crucial for implementing appropriate clinical management and achieving optimal patient outcomes.
